# Growth, Pathogenesis, and Serological Characteristics of the Japanese Encephalitis Virus Genotype IV Recent Strain 19CxBa-83-Cv

**DOI:** 10.3390/v15010239

**Published:** 2023-01-14

**Authors:** Shigeru Tajima, Takahiro Maeki, Eri Nakayama, Astri Nur Faizah, Daisuke Kobayashi, Haruhiko Isawa, Yoshihide Maekawa, Sri Subekti Bendryman, Kris Cahyo Mulyatno, Etik Ainun Rohmah, Yasuko Mori, Kyoko Sawabe, Hideki Ebihara, Chang-Kweng Lim

**Affiliations:** 1 Department of Virology 1, National Institute of Infectious Diseases, 1-23-1 Toyama, Shinjuku, Tokyo 162-8640, Japan; 2 Department of Medical Entomology, National Institute of Infectious Diseases, 1-23-1 Toyama, Shinjuku, Tokyo 162-8640, Japan; 3 Laboratory of Entomology, Institute of Tropical Diseases, Universitas Airlangga, Surabaya 60115, Indonesia; 4 Division of Clinical Virology, Center for Infectious Diseases, Kobe University Graduate School of Medicine, Kobe 650-0017, Japan

**Keywords:** Japanese encephalitis virus, genotype IV, pathogenicity, serology

## Abstract

Genotype IV Japanese encephalitis (JE) virus (GIV JEV) is the least common and most neglected genotype in JEV. We evaluated the growth and pathogenic potential of the GIV strain 19CxBa-83-Cv, which was isolated from a mosquito pool in Bali, Indonesia, in 2019, and serological analyses were also conducted. The growth ability of 19CxBa-83-Cv in Vero cells was intermediate between that of the genotype I (GI) strain Mie/41/2002 and the genotype V (GV) strain Muar, whereas 19CxBa-83-Cv and Mie/41/2002 grew faster than Muar in mouse neuroblastoma cells. The neuroinvasiveness of 19CxBa-83-Cv in mice was higher than that of Mie/41/2002 but lower than that of Muar; however, there were no significant differences in neurovirulence in mice among the three strains. The neutralizing titers of sera from 19CxBa-83-Cv- and Mie/41/2002-inoculated mice against 19CxBa-83-Cv and Mie/41/2002 were similar, whereas the titers against Muar were lower than those of the other two viruses. The neutralizing titers of JE vaccine-inoculated mouse pool serum against 19CxBa-83-Cv and Muar were significantly lower than those against Mie/41/2002. The neutralizing titers against the three viruses were similar in three out of the five serum samples from GI-infected JE patients, although the titers against Mie/41/2002 were higher than those against 19CxBa-83-Cv and Muar in the remaining two sera samples. In summary, we identified the basic characteristics of 19CxBa-83-Cv, but further studies are needed to better understand GIV JEV.

## 1. Introduction

Japanese encephalitis (JE) is a serious nervous disorder and is a significant public health problem in many Asian countries. Humans are infected with JEV by being bitten mainly by *Culex* mosquitoes. There are an estimated 68,000 cases of JE per year in East, South, and Southeast Asian countries, resulting in 15,000 fatalities, mostly in children [1,2,3]. There is no specific treatment available for JE other than preventive vaccination with JE vaccines. 

JEV belongs to the genus Flavivirus in the family Flaviviridae and is amplified in bird and pig–mosquito transmission cycles [4]. Ardeid birds are the natural reservoir for JEV, whereas pigs act as the major amplifying hosts of JEV and are mainly involved in outbreaks in humans. Infected Culex mosquitoes transmit JEV to dead-end hosts, either humans or horses. JEV has a single-stranded positive-sense RNA genome that encodes three structural proteins (C, prM, and E) and seven non-structural proteins (NS1, NS2A, NS2B, NS3, NS4A, NS4B, and NS5). The genome also contains non-coding regions (NCRs) at the 5′- and 3′-terminal ends. JEV is classified into five genotypes (GI-GV), and the Indonesian/Malaysian region is thought to be the origin of all genotypes of JEV [5]. GIII was the most widely distributed and frequently isolated in JE-endemic areas until the 1990s. In recent years, the most commonly isolated genotype of JEV has changed from GIII to GI strains in most JE-endemic areas [6,7,8,9,10,11,12,13,14,15,16]. Although the reason for this broad shift remains unclear, GI strains circulating in recent years might replicate more efficiently in birds and mosquitoes [17,18]. GII strains have been found in Indonesia, Singapore, Korea, Malaysia, and Australia [19,20]. GV JEV, which was first identified in Malaysia in the 1950s, was detected for the first time in over 50 years in China in 2009 and in Korea in 2010 [21,22]. Furthermore, GV is the major JEV strain currently detected in Korea [23,24]. Several groups have attempted to elucidate the growth and pathogenic properties of GV JEV, and their findings suggest that GV JEV has growth and pathogenic characteristics that differ from those of the major genotypes GI and GIII [25,26,27,28,29]. GIV JEV is the least common genotype. GIV strains were found in mosquitoes from Java, Bali, and Flores Island in Indonesia in the 1980s [5,19]. Given that only a few GIV strains have been isolated, biological studies on the features of GIV JEV are limited, and the growth and pathogenicity of GIV remain poorly understood. However, GIV strains were isolated from mosquitoes and pigs in Bali in 2017 and 2019 [20,30] and from an Australian JE patient who traveled from Bali in 2019 [31]. 

In Australia, JEV infection in humans was first identified in the Torres Strait Islands in the state of Queensland in 1995 [32]. The nucleotide sequence of JEV isolated from asymptomatic human residents in the area and mosquito samples indicated that the genotype of the virus was GII [32,33,34]. The next JE epidemic occurred in the Torres Strait Islands and mainland Australia in 1998 [35]. Since 1995, the transmission of JEV has occasionally been confirmed in mosquitoes and pigs in northern Australia [36]. Genetic analysis of the JEV isolates from mosquitoes and pigs indicated that the isolates in 1998 also belonged to GII, whereas the isolate from Badu in the Torres Strait Islands in 2004 belonged to GI [36,37]. A JE patient who was thought to be infected with GIV JEV in the Tiwi islands in northern Australia was confirmed as infected in Australia in 2021 following a 20-year absence of JE [38,39,40]. In 2022, JEV was widespread in Australia. During the outbreak, JE patients were confirmed in five states/territories in mainland Australia, where JEV was not endemic [41]. As of 22 October 2022, over 40 human JE cases, with seven fatal cases, have been reported [41]. The outbreak of JEV was first identified in swine and subsequently in humans, mosquitoes, and sentinel chickens [42]. Genetic analysis of the virus associated with the epidemics in 2021 and 2022 indicated that the virus was GIV JEV [38,40,43]. 

To elucidate the basic characteristics of this neglected genotype of JEV, we investigated the in vitro growth and virulence of the GIV JEV strain 19CxBa-83-Cv, which was isolated from *Culex vishnui* collected in Bali in 2019 [30]. Furthermore, the neutralizing efficacy of mouse serum immunized with inactivated JE vaccines and human sera from JE patients against the 19CxBa-83-Cv strain as well as GI and GIII strains was also investigated to explore the serological relationship among JEV genotypes.

## 2. Materials and Methods

### 2.1. Viruses

The GIV strain 19CxBa-83-Cv (GenBank accession No.LC579814) was isolated from a pool of the mosquito *Culex vishunui* collected in Bali, Indonesia, in 2019 using mosquito-derived C6/36 cells [30]. The GI JEV strain Mie/41/2002 (GenBank accession No. AB241119) and GV strain Muar (GenBank accession No. HM59272) were also used [27]. The working virus stocks were prepared by amplification in Vero cells. 

### 2.2. Cell Culture

African green monkey kidney Vero cells (strain 9013), human neuroblastoma IMR-32 cells, and mouse neuroblastoma Neuro-2a cells were cultured at 37 °C in 5% CO_2_ in Eagle’s minimal essential medium (MEM) (Sigma-Aldrich, St. Louis, MO, USA) supplemented with 10% heat-inactivated fetal bovine serum (FBS) (CORNING, Corning, NY, USA) and 100 U/mL penicillin-streptomycin (Nacalai Tesque, Kyoto, Japan).

### 2.3. Phylogenetic Analysis

The phylogenetic tree was constructed with the maximum likelihood method using 500 bootstrap replications (Molecular Evolutionary Genetics Analysis software; MEGA X) based on the complete nucleotide sequence (1500 nucleotides) of the JEV E gene [44].

### 2.4. Plaque Formation Assay for Titration of Infectious Viruses and Analysis of Growth Kinetics 

Infectious viral titers for each sample were determined using plaque formation assays. Vero cells (approximately 5 × 10^5^/well) were plated in 12-well culture plates and inoculated with each virus for 1 h at 37 °C. Next, an MEM-based overlay medium containing 1% methylcellulose (FUJIFILM Wako Pure Chemical, Osaka, Japan) and 2% FBS was added to the wells, and the cells were incubated for 4–5 days at 36 °C, after which the cells were fixed using a 10% formalin–PBS solution and stained with methylene blue, as described previously [45]. The diameter of 20 plaques was measured and the mean plaque size in mm ±SEM was calculated. Differences in mean plaque sizes were analyzed using Welch’s t-test. The ability of the JEV strains to grow in vitro was analyzed as previously described [46]. Briefly, cells were cultured in 6-well culture plates and infected with each JEV strain in 3 mL MEM supplemented with 2% FBS (2F/MEM) at a multiplicity of infection (MOI) of 0.02–0.1 plaque-forming units (PFU)/cell. Small aliquots (200 μL) of the media were collected at one-day intervals, and infectious viral titers were determined by a plaque formation assay in Vero cells, as described above. 

### 2.5. Mouse Challenge Experiment

Female ddY mice (Japan SLC, Shizuoka, Japan) were used for the challenge tests. For neuroinvasive analysis, groups of mice (3 weeks old, *n* = 6) were inoculated intraperitoneally (i.p.) with 100 μL (1 × 10^3^, 1 × 10^4^, or 1 × 10^5^ PFU) of virus solution diluted in 0.9% NaCl solution. The mice were observed, and their body weights were measured every day for 20 days after inoculation to assess survival rates. Survival curves were compared using Bell Curve for Excel (Social Survey Research Information, Tokyo, Japan) and the log-rank (Mantel–Cox) test. Statistical significance was set at *p* < 0.05. The surviving mice were sacrificed, and their sera were collected for serological neutralization tests, as described in Section 2.8. For analysis of neurovirulence, groups of mice (4 weeks old, *n* = 6) were inoculated intracerebrally (i.c.) with 30 μL (3 × 10^2^ or 3 × 10^3^ PFU) of virus solution, and the mice were observed for 14 days to determine survival rates, as described above.

### 2.6. Mouse Vaccination

Female ddY mice (4 weeks old) were vaccinated i.p. with 0.5 mL of Vero cell-derived inactivated JE Beijing-1 (GIII) vaccine (BIKEN: Lot no. 106VC-2009) and were immunized again one week after the initial immunization. The immunized mice were sacrificed 14 days after the initial immunization, and their sera were collected and mixed. The pooled serum solution (JSS-2020) was used for the neutralization test, as described in Section 2.8. 

### 2.7. Sera of JE Patients

Sera from four JE patients in Japan between 2019 and 2021 were also used for the neutralization study. The patients were diagnosed with JE in our laboratory using JEV-specific IgM ELISA and neutralization tests, as described previously [47]. The JEV genotypes were determined by conventional RT-PCR amplification of the JEV E region, followed by nucleotide sequencing, as described previously [48]. 

### 2.8. Plaque Reduction Neutralization Test (PRNT)

Neutralizing antibodies against JEV were measured using the PRNT method. Each JEV strain was combined at a 1:1 ratio with 2-fold serial dilutions (1:10 to 1:10,240) of human and mouse sera, and then incubated at 37 °C for 90 min. Vero cell monolayers were inoculated with the mixtures in 12-well plates and incubated at 37 °C for 90 min. Subsequently, an overlay medium containing 1% methylcellulose was added, and cells were incubated at 36 °C for 4–5 days. The cells were fixed using a 10% formalin–PBS solution and stained with methylene blue. The PRNT titer (PRNT_50_) was defined as the reciprocal of the highest dilution resulting in a 50% reduction relative to the mouse serum-free control.

## 3. Results

### 3.1. Phylogenetic Analysis of the 19CxBa-83-Cv Strain

**Figure 1:**
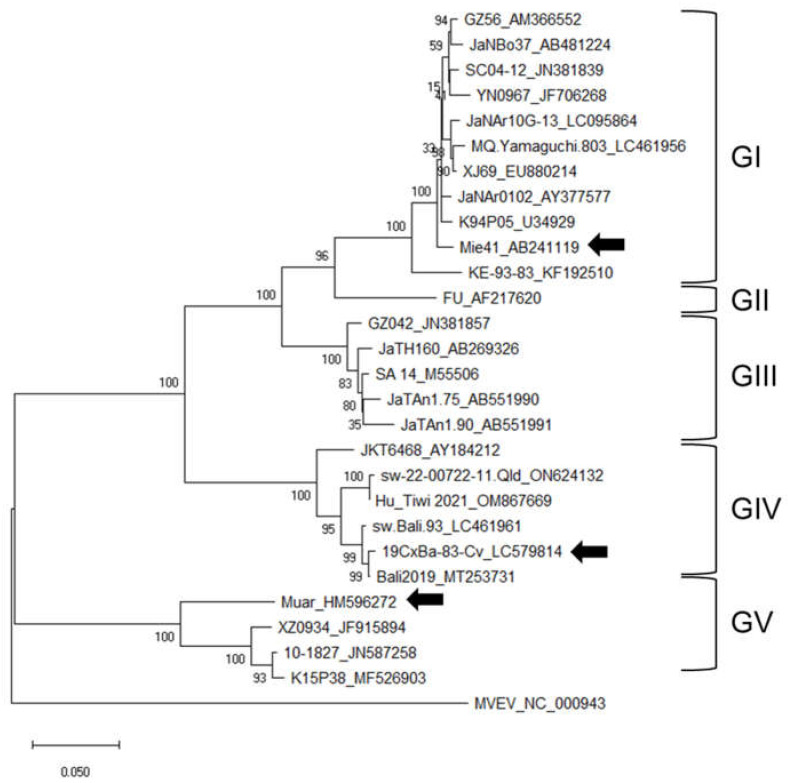
Phylogenetic tree of JEV based on the complete nucleotide sequences of the E gene. Numbers at branch points indicate bootstrap values (500 replicates). Genotypes (GI, GII, GIII, GIV, and GV) are shown. Arrowheads indicate the three strains (GI Mie/41/2002, GIV 19CxBa-83-Cv, and GV Muar) that were characterized further in this study.

A phylogenetic tree of JEV was constructed using the nucleotide sequence of the E gene of the GI–GV JEV strains (Figure 1). Strain 19CxBa-83-Cv was clustered in the GIV and showed higher homology to the Bali2019 strain isolated from an Australian JE patient infected in Bali in 2019, compared to the strains JEV/Aus/NT_Tiwi Islands/2021 (Hu_Tiwi 2021; OM867669) and sw-22-00722-11 (ON624132) identified in Australia in 2021 and 2022, respectively [30,31,38,40]. 

### 3.2. Growth Properties of the 19CxBa-83-Cv Strain In Vitro

**Figure 2:**
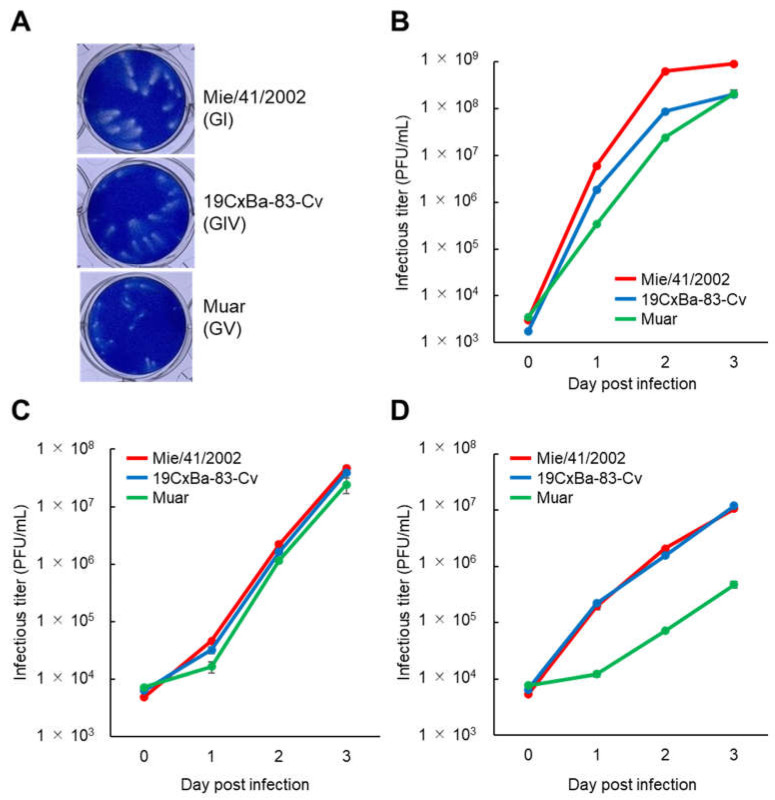
Growth properties of the GI, GIV, and GV JEV strains. (**A**) Plaque phenotypes of GI Mie/41/2002, GIV 19CxBa-83-Cv, and GV Muar in Vero cells. (**B**–**D**) Growth curves were measured for Mie/41/2002, 19CxBa-83-Cv, and Muar in Vero (**B**), IMR-32 (**C**), and Neuro-2a (**D**). Cells were plated into 6-well culture plates and infected with the viruses at a multiplicity of infection (MOI) of 0.02 (Vero) or 0.1 (IMR-32, Neuro-2a) plaque forming units (PFU)/cell. Values: means ± standard deviation from three independent inoculations.

To clarify the growth properties of the GIV strain 19CxBa-83-Cv in vitro, we first compared the plaques formed by infection with 19CxBa-83-Cv to those of the GI strain Mie/41/2002 and GV strain Muar in Vero cells (Figure 2A). The plaque size induced by 19CxBa-83-Cv (mean diameter ±SEM: 0.994 ± 0.046) was smaller than that induced by Mie/41/2002 (1.313 ± 0.049; *P* < 0.001) but was larger than that induced by Muar (0.671 ± 0.020; *P* < 0.001). Analysis of the growth kinetics of the strains showed that 19CxBa-83-Cv grew slower than Mie/41/2002, whereas 19CxBa-83-Cv grew faster than Muar in Vero cells (Figure 2B). The growth rate of 19CxBa-83-Cv was similar to those of Mie/41/2002 and Muar in human neuroblastoma IMR-32 cells (Figure 2C). We previously showed that the growth ability of the Muar strain was lower than that of Mie/41/2002 in murine neuroblastoma cells [27,29]. The growth rate of 19CxBa-83-Cv was quite close to that of Mie/41/2002 and strikingly higher than that of Muar in Neuro-2a cells (Figure 2D). 

### 3.3. Virulence of the 19CxBa-83-Cv Strain in Mice

**Figure 3:**
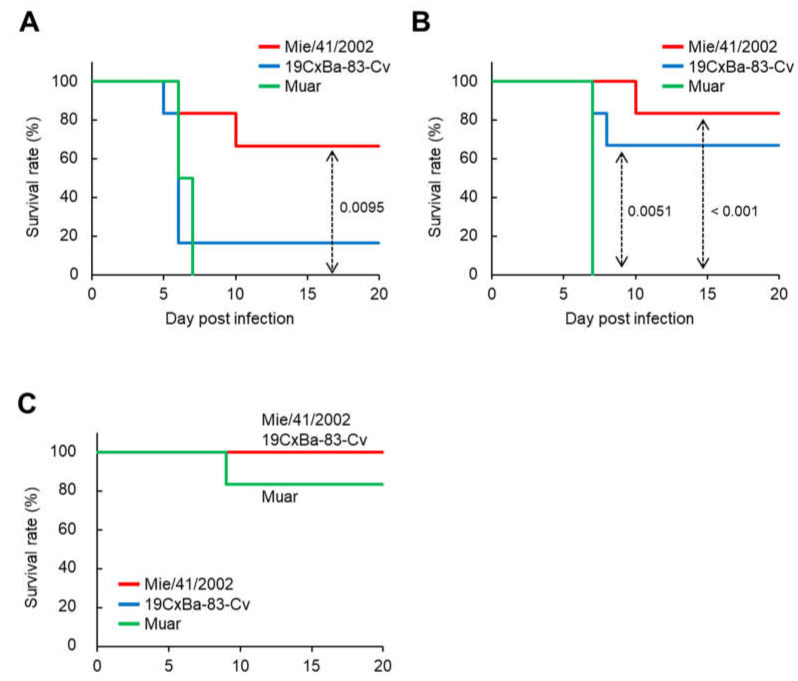
Murine neuroinvasiveness of the GI, GIV, and GV JEV strains. Survival curve of mice inoculated intraperitoneally with 1 × 105 PFU (**A**), 1 × 104 PFU (**B**), and 1 × 103 PFU (**C**) of Mie/41/2002 (*n* = 6), 19CxBa-83-Cv (*n* = 6), or Muar (*n* = 6). Significant p values by the log-rank test are also indicated.

Next, we examined the neuroinvasiveness of 19CxBa-83-Cv in mice. Mice were infected intraperitoneally with 19CxBa-83-Cv, Mie/41/2002, or Muar, and their body weights were recorded (Figure 3 and Figure S1). We previously reported that the neuroinvasiveness of Muar was significantly higher than that of Mie/41/2002 in mice, even though Muar grew slower than Mie/41/2002 in murine neuroblastoma cells, as shown in Figure 2D [27]. In the group infected with 10^5^ PFU, all the mice infected with Muar died, and five out of six (83.3%) 19Cx-83-Cv-infected mice died by seven days after inoculation, although only two out of six (33.3%) Mie/41/2002-infected mice died by 10 days post-infection (Figure 3A). The survival curve for the 19CxBa-83-Cv-infected group was similar to that of the Muar-infected group. In the group infected with 10^4^ PFU, two (33.3%) and one (16.7%) out of six 19CxBa-83-Cv-infected mice and six Mie/41/2002-infected mice died within 10 days post-infection, respectively, whereas all Muar-infected mice died by seven days post-infection (Figure 3B). In the group infected with 10^3^ PFU, all mice infected with the 19CxBa-83-Cv or Mie/41/2002 survived, but one out of six (16.7%) Muar-infected mice died throughout the observation period (Figure 3C). The survival curves of the 19CxBa-83-Cv-infected groups were similar to those of the Mie/41/2002-infected groups in mice infected with 10^4^ PFU and 10^3^ PFU. 

**Figure 4:**
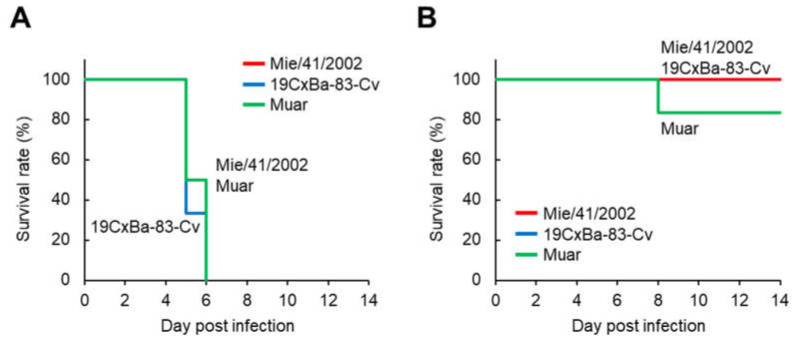
Murine neurovirulence of the GI, GIV, and GV JEV strains. Survival curve of mice inoculated intracerebrally with 3 × 10^3^ PFU (**A**) and 3 × 10^2^ PFU (**B**) of Mie/41/2002 (*n* = 6), 19CxBa-83-Cv (*n* = 6), or Muar (*n* = 6).

To evaluate the neurovirulence of 19CxBa-83-Cv in mice, the mice were infected i.c. with 19CxBa-83-Cv, Mie/41/2002, or Muar, and their body weights were recorded (Figure 4 and Figure S2). In the groups infected with 3 × 10^3^ PFU, all mice infected with 19CxBa-83-Cv, Mie/41/2002, or Muar died within six days after infection (Figure 4A). In contrast, all mice infected with 3 × 10^2^ PFU of 19CxBa-83-Cv or Mie/41/2002 survived, and one out of six (16.7%) mice infected with Muar died throughout the observation period (Figure 4B). No significant differences were observed in the survival curves among the groups infected with 3 × 10^3^ PFU and those infected with 3 × 10^2^ PFU. 

### 3.4. Neutralizing Ability of Sera from the 19CxBa-83-Cv-Infected Mice

Serum samples were collected from the surviving mice at the end of the observation period (20 days post-infection) in the neuroinvasive experiment described in Figure 3. The sera were subjected to neutralization analysis against 19CxBa-83-Cv, Mie/41/2002, and Muar (Table 1). Sera from mice inoculated with 10^5^ or 10^4^ PFU 19CxBa-83-Cv showed PRNT_50_ titers between 1:160 and 1:640 against 19CxBa-83-Cv and between 1:160 and 1:1280 against Mie/41/2002, whereas the sera showed lower titers against Muar (1:40–1:160). Sera from 10^5^ or 10^4^ PFU Mie/41/2002-infected mice exhibited a 1:640 or 1:1280 ratio against Mie/41/2002, a 1:320 ratio against 19CxBa-83-Cv, and a 1:80 or 1:160 ratio against Muar. All sera from 10^3^ PFU-infected mice showed low titers of all three viruses (1:10 or <1:10). 

### 3.5. Neutralizing Ability of Pooled Serum from Mice Vaccinated with Vero Cell-Derived Inactivated JE Vaccine against the 19CxBa-83-Cv

We wished to evaluate the ability of the Vero cell-derived inactivated JE vaccine, which was produced from the GIII JEV Beijing-1 strain in Japan, to induce neutralizing antibodies against 19CxBa-83-Cv. Pooled mouse serum JSS-2020, which was prepared from mice immunized twice with the vaccine, was used for PRNT against 19CxBa-83-Cv, Mie/41/2002, and Muar (Table 2). The PRNT_50_ titer of the serum against Mie/41/2002 was 1:160, which was higher than those against 19CxBa-83-Cv (1:20) and Muar (1:40). 

### 3.6. Neutralizing Ability of Sera from JE Patients against 19CxBa-83-Cv

Five sera samples from four autochthonous JE patients were collected to assess the neutralizing ability against 19Cx-Ba-83-Cv, Mie/41/2002, and Muar (Table 3). The nucleotide sequences of the E region of the JEV genome amplified from the samples of the patients indicated that all JE patients were infected with GI JEV. The PRNT_50_ titers against the three viruses were similar in three of the five sera. In the remaining two sera, the titers against GI Mie/41/2002 were four- to sixteen-fold higher than those against GIV 19CxBa-83-Cv and GV Muar.

## 4. Discussion

The first case of JE in humans caused by GIV JEV infection was reported in 2019 [31]. The endemics of JE in Australia in 2021 and 2022 were also caused by GIV JEV [38,39,40]. However, only a few GIV JEV have been isolated before the epidemics, little attention has been paid to GIV JEV, and GIV JEV has not been well characterized. In this study, we characterized the recently isolated GIV JEV strain 19CxBa-83-Cv [30].

The phylogenetic tree in Figure 1 shows that the 2021–2022 Australian strains were located between the 2017–2019 Bali group, which contains 19CxBa-83-Cv, and the 1980s Indonesian group (JKT6468), suggesting that the Australian endemic strains may not originate from the recent Bali group. The E protein of 19CxBa-83-Cv differs by only one amino acid residue (0.2%) from that of the Bali2019 strain, which was isolated from an Australian JE patient returning from Bali in 2019, whereas the strain differs by eight residues (1.6%) from that of the 2021–2022 Australian strains (Figure S3). The Australian strains also have some residues unique to the group in the E protein [40]. Further genomic analyses of more GIV strains will help us to understand the meaning of the variations in amino acid sequences among GIV strains. 

The growth potential and plaque size of 19CxBa-83-Cv in Vero cells were intermediate between those of GI strain Mie/41/2002 and GV strain Muar. The three strains showed similar growth properties in human neuroblastoma-derived IMR-32 cells, whereas Muar grew notably slower than the other two strains, which showed similar growth patterns in mouse neuroblastoma Neuro-2a cells. We previously reported that Muar exhibits slower growth kinetics than Mie/41/2002 in mouse neuroblastoma cells and that an amino acid residue in the non-structural protein NS2A (NS2A^166^) is crucial for the growth of GI and GV JEV in the cells [27,29]. The residue NS2A^166^ in Muar is His, but in Mie/41/2002 and 19CxBa-83-Cv, it is Tyr, suggesting that the Tyr residue NS2A^166^ in 19CxBa-83-Cv contributes to the efficient replication ability of 19CxBa-83-Cv in Neuro-2a. 

The neuroinvasiveness of 19CxBa-83-Cv in mice was higher than that of Mie/41/2002 but lower than that of Muar. It is known that the E protein of JEV is the main factor determining its pathogenicity [49,50,51,52]. Our previous findings indicate that the amino acid at position 123 in the E protein (E^123^) is involved in the neuroinvasiveness of JEV in mice. JEV with Ser at E123 shows lower neuroinvasiveness than JEV with Arg and His at this position [28,45]. His at E^123^ is highly conserved in GV JEV, including Muar, whereas Mie/41/2002 and 19CxBa-83-Cv have Ser at this position (Figure S4). In our results, 19CxBa-83-Cv exhibited higher neuroinvasiveness in mice than Mie/41/2002, indicating that E123 is not associated with 19CxBa-83-Cv in neuro-invasiveness in mice. Twenty-three residues in the E protein differed between 19CxBa-83-Cv and Mie/41/2002, and these sites may include amino acid residues responsible for the difference in pathogenicity between the strains. Alternatively, it is also possible that viral proteins other than the E protein are involved in the variation in virulence. The establishment of reverse genetics for GIV JEV is needed to analyze the pathological mechanisms of GIV JEV. There were no significant differences in neurovirulence in mice among the three strains, implying that the direct ability of the strains to damage the brain is not involved in the differences in neuro-invasiveness among the viruses. The ability of the strains to proliferate in the peripheral tissues of mice may be important for determining the pathogenicity of the viruses. 

The neutralizing titers of sera from the 19CxBa-83-Cv- and Mie/41/2002-inoculated mice against 19CxBa-83-Cv and Mie/41/2002 were similar, whereas the titers against Muar were four-fold lower than those against the other two viruses in most mice. These data suggest that 19CxBa-83-Cv is serologically closer to the GI strain Mie/41/2002 than to GV Muar. A comparison of the amino acid sequences of the E protein showed that the identity between 19CxBa-83-Cv and Mie/41/2002 was 95.4%, whereas that between 19CxBa-83-Cv and Muar was 90.6% (Figure S4), supporting the idea that 19CxBa-83-Cv is closer to Mie/41/2002 than to Muar. 

GIII strains were widely and frequently isolated in most JE endemic areas until the 1990s; therefore, licensed JE vaccines are produced from GIII strains [53,54,55]. A previous report indicated that the chimeric JE vaccine induced protective levels of neutralizing antibodies against the classical GIV strain 9092 [56]. We investigated the neutralizing potency of the GIII Beijing-1-based inactivated JE vaccine against the three strains using mice and found that the titers of pooled sera from vaccinated mice against 19CxBa-83-Cv and Muar were lower than those against Mie/41/2002. In the E protein, there were only eight residues (1.6%) that differed between Beijing-1 and Mie/41/2002, but 26 residues (5.2%) and 42 residues (8.4%) differed between Beijing-1 and 19CxBa-83-Cv and between Beijing-1 and Muar, respectively (Figure S4), implying that the differences in the amino acid sequences of the structural proteins may influence the neutralization potency of JE vaccines. 

The major genotype of JEV isolated in Japan is GI [48,57,58]. In this study, we used five serum samples from four Japanese JE patients who were infected with GI JEV in Japan to evaluate the reactivity of the sera to the three strains. In three of the five serum samples, no clear differences in the neutralizing titers against the strain were observed. In contrast, in the other two serum samples, the titers against 19CxBa-83-Cv and Muar were four- to sixteen-fold lower than those against Mie/41/2002. Our previous reports showed that the ratio of the neutralizing titer against Muar to that against Mie/41/2002 was equivalent to or less than 1:2 in most JE patient sera in Japan and northern Vietnam, where only GI JEV has been identified in recent years [59,60]. These findings are partially inconsistent with the data from JEV-infected mouse samples described above. The reasons for this remain unknown. 

In this study, we uncovered the basic characteristics of the recent GIV JEV strain 19CxBa-83-Cv by comparing the virus with well-characterized GI and GV JEV strains. However, there are certain differences in the amino acid sequences between the 2017–2019 Bali group and the 2021–2022 Australian group, which might critically affect the properties of the viruses. Therefore, it is essential to conduct similar analyses of other GIV isolates and accumulate information on the isolates in the future to understand the nature of GIV JEV.

## Figures and Tables

**Table 1:** Neutralization titers (PRNT_50_) in sera of mice infected intraperitoneally with JEV strains Mie/41/2002, 19CxBa-83-Cv, and Muar against the JEVs ^1^.

Mouse	Titer Inoculated	Mouse ID	PRNT_50_ against:		
			Mie/41/2002 (GI)	19CxBa-83-Cv (GIV)	Muar (GV)
Mie/41/2002-inoculated group	10^5^ 10^4^ 10^4^ 10^3^	PR PY PYG PW	640 1280 640 <10	320 320 320 <10	80 80 160 <10
19CxBa-83-Cv-inoculated group	10^5^ 10^4^ 10^4^ 10^4^ 10^3^	YG YW YB YG YW	1280 320 320 160 10	640 160 320 320 <10	160 40 80 40 <10
Muar-inoculated group	10^3^	GW	<10	<10	<10

^1^ Sera were collected from the survived mice at 20 days after inoculation of the JEVs described in Figure 3.

**Table 2:** Neutralization titers (PRNT_50_) in serum of mice vaccinated with formalin-inactivated JE vaccine against JEV strains Mie/41/2002, 19CxBa-83-Cv, and Muar.

Serum	PRNT_50_ against:		
	Mie/41/2002 (GI)	19CxBa-83-Cv (GIV)	Muar (GV)
JSS-2020 ^1^	160	20	40

^1^ Mouse sera which were recovered from mice immunized twice with the inactivated JE vaccine (GIII Beijing-1 strain, Lot 106-2009) and mixed.

**Table 3:** Neutralization titers (PRNT_50_) in sera from GI JEV-infected JE patients against JEV strains Mie/41/2002, 19CxBa-83-Cv, and Muar ^1^.

Patient	Days after Onset	PRNT_50_ against:		
		Mie/41/2002 (GI)	19CxBa-83-Cv (GIV)	Muar (GV)
1	25	2560	2560	2560
2	7	160	80	80
	16	5120	5120	5120
3	10	640	160	160
4	10	2560	320	160

^1^ Sera were collected from four domestic JE patients diagnosed by IgM ELISA, real-time RT-PCR, and PRNT_50_ methods.

## Data Availability

The data presented in this study are available on request from the corresponding author.

## Supplementary Materials

The following supporting information can be downloaded at: https://www.mdpi.com/article/10.3390/v15010239/s1, Figure S1: Body weight of mice inoculated with JEV strains as shown in Figure 3. Figure S2: Body weight of mice inoculated with JEV strains as shown in Figure 4. Figure S3: Comparison of the complete amino acid sequence of E protein (500 residues) in GIV JEV strains. Figure S4: Comparison of the complete amino acid sequence of E protein (500 residues) of Mie/41/2002 (GI), Beijing-1 (GIII), 19CxBa-83-Cv (GIV), and Muar (GV) strains.
