# Supplementary material for: Growth, Pathogenesis, and Serological Characteristics of the Japanese Encephalitis Virus Genotype IV Recent Strain 19CxBa-83-Cv
Source: Viruses. 2023 Jan 14;15(1):239. doi: 10.3390/v15010239 (PMC9866982; doi:10.3390/v15010239)
Supplement: Supplementary file 1 [file viruses-15-00239-s001.zip › viruses-2143271-supplementary.pdf]

Figure S1

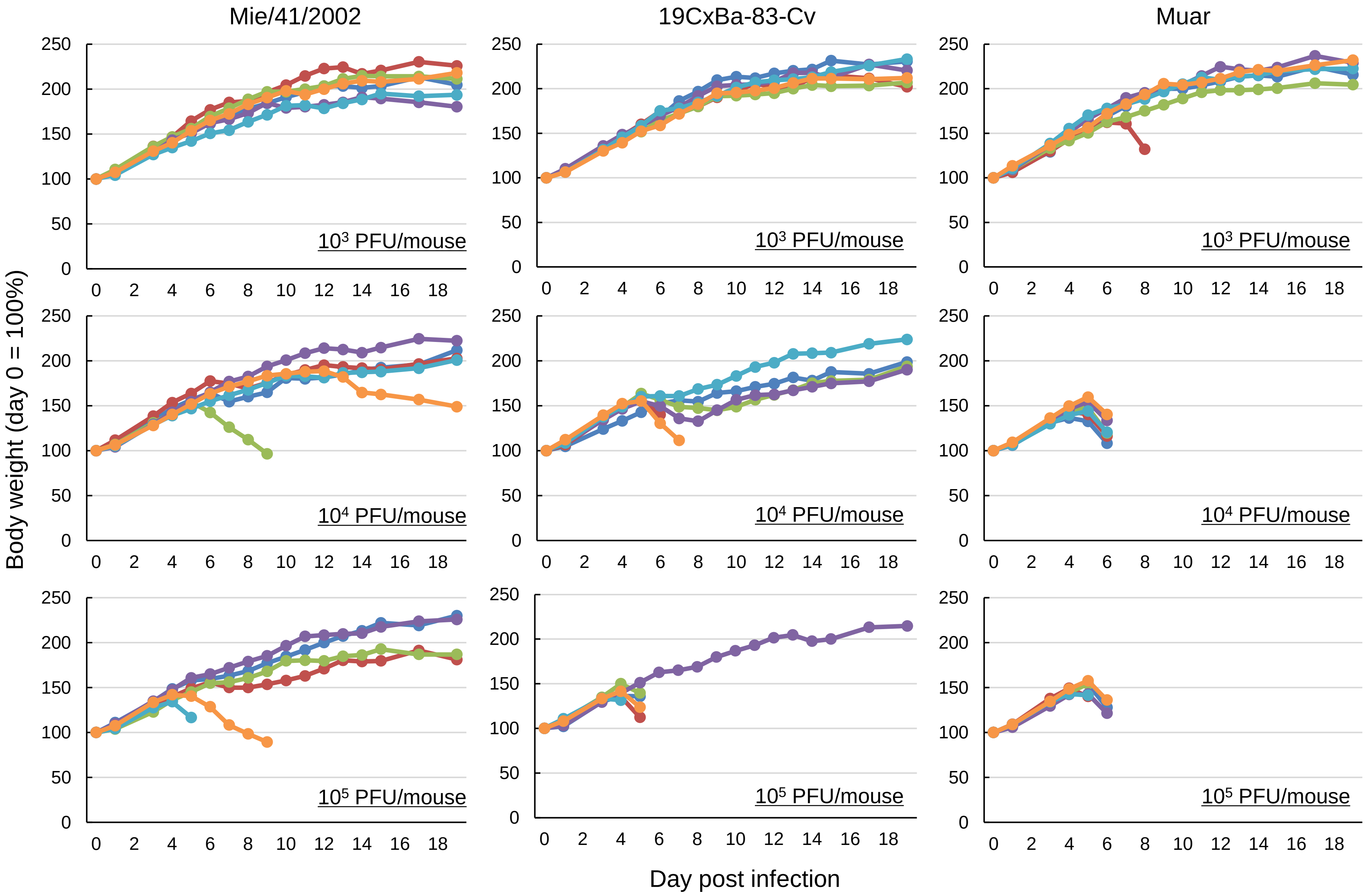

Figure S1. Body weight of mice inoculated with JEV strains (GI, GIV, GV) at three different doses (10^3, 10^4, 10^5 PFU/mouse) as shown in Figure 3. Colored lines indicate individual mice.

Figure S2

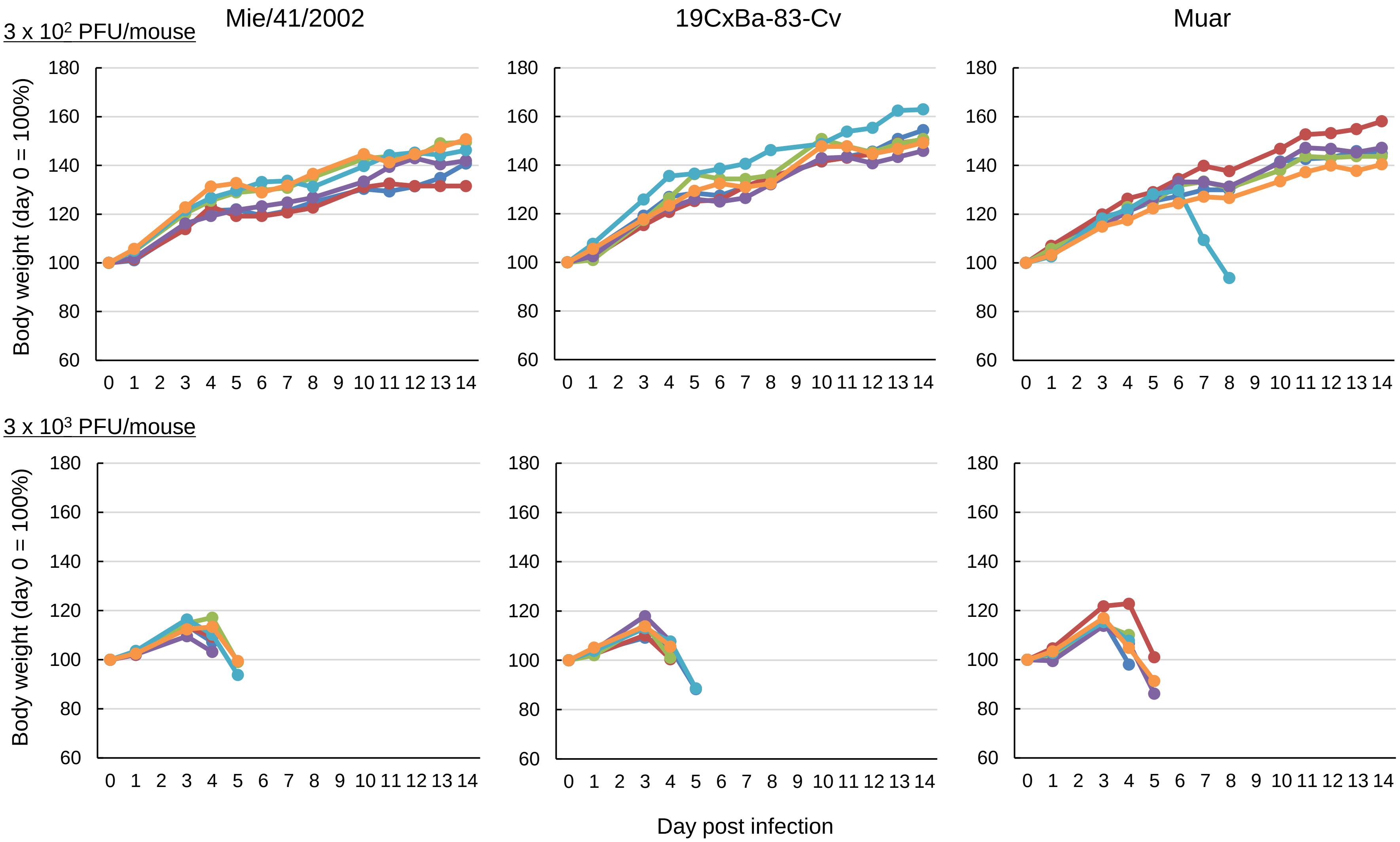

Figure S2. Body weight of mice inoculated with JEV strains (GI, GIV, GV) at two different doses (3 x 10^2, 3 x 10^3 PFU/mouse) as shown in Figure 4. Colored lines indicate individual mice.

Figure S3

```
19CxBa-83-Cv_LC579814       1 FNCLGMGNRDFIEGVSGATWVDLVLEGDSCLTIMAHDRPTLDVRMVNIEASQLAEVRSYCYHASVTDISTVARCPTTGEAHNDKRADSNYVCKQGFTDRG 100
sw-22-00722-11.Qld_ON624132 1 FNCLGMGNRDFIEGVSGATWVDLVLEGDSCLTIMAHDRPTLDVRMINIEASQLAEVRSYCYHASVTDISTVARCPTTGEAHNEKRADSSYVCKQGFTDRG 100
Hu_Tiwi 2021_OM867669       1 FNCLGMGNRDFIEGVSGATWVDLVLEGDSCLTIMAHDRPTLDVRMINIEASQLAEVRSYCYHASVTDISTVARCPTTGEAHNEKRADSSYVCKQGFTDRG 100
Bali2019_MT253731           1 FNCLGMGNRDFIEGVSGATWVDLVLEGDSCLTIMAHDRPTLDVRMINIEASQLAEVRSYCYHASVTDISTVARCPTTGEAHNDKRADSNYVCKQGFTDRG 100
sw.Bali.93_LC461961         1 FNCLGMGNRDFIEGVSGATWVDLVLEGDSCLTIMAHDRPTLDVRMINIEASQLAEVRSYCYHASVTDISTVARCPTTGEAHNDKRADSNYVCKQGFTDRG 100
JKT6468_AY184212            1 FNCLGMGNRDFIEGVSGATWVDLVLEGDSCLTIMANDRPTLDVRMINIEASQLAEVRSYCYHASVTDISTVARCPTTGEAHNEKRADSSYVCKQGFTDRG 100

19CxBa-83-Cv_LC579814     101 WGNGCGLFGKGSIDTCAKFSCTSKATGKTIQPENIKYEVGIFVHGTTTSENHGNYTAQIGASQAAKFTITPNAPSITLKLGDYGEVTLDCEPRSGLNTEA 200
sw-22-00722-11.Qld_ON624132 101 WGNGCGLFGKGSIDTCAKFSCTSKAIGKTIQPENIKYEVGVFVHGTTTSENHGNYTAQIGASQAAKFTITPNAPSITLKLGDYGEVTLDCEPRNGLNTEA 200
Hu_Tiwi 2021_OM867669     101 WGNGCGLFGKGSIDTCAKFSCTSKAIGKTIQPENIKYEVGVFVHGTTTSENHGNYTAQIGASQAAKFTITPNAPSITLKLGDYGEVTLDCEPRNGLNTEA 200
Bali2019_MT253731         101 WGNGCGLFGKGSIDTCAKFSCTSKATGKTIQPENIKYEVGIFVHGTTTSENHGNYTAQIGASQAAKFTITPNAPSITLKLGDYGEVTLDCEPRSGLNTEA 200
sw.Bali.93_LC461961       101 WGNGCGLFGKGSIDTCAKFSCTSKATGKTIQPENIKYEVGIFVHGTTTSENHGNYTAQIGASQAAKFTITPNAPSITLKLGDYGEVTLDCEPRSGLNTEA 200
JKT6468_AY184212          101 WGNGCGLFGKGSIDTCAKFSCTSKATGKTIQPENIKYEVGIFVHGTTTSENHGNYTAQIGASQAAKFTITPNAPSITLKLGDYGEVTLDCEPRSGLNTEA 200

19CxBa-83-Cv_LC579814     201 FYVMTVGSKSFLVHREWFHDLALPWTSASNTAWRNRELLMEFEEAHATKQSVVALGSQEGALHQALAGAIVVEYSSSVKLTSGHLKCRLKMDKLTLKGTT 300
sw-22-00722-11.Qld_ON624132 201 FYVMTVGSKSFLVHREWFHDLALPWTSSSNTAWRNRELLMEFEEAHATKQSVVALGSQEGALHQALAGAIVVEYSSSVKLTSGHLKCRLKMDKLTLKGTT 300
Hu_Tiwi 2021_OM867669     201 FYVMTVGSKSFLVHREWFHDLALPWTSSSNTAWRNRELLMEFEEAHATKQSVVALGSQEGALHQALAGAIVVEYSSSVKLTSGHLKCRLKMDKLTLKGTT 300
Bali2019_MT253731         201 FYVMTVGSKSFLVHREWFHDLALPWTSSSNTAWRNRELLMEFEEAHATKQSVVALGSQEGALHQALAGAIVVEYSSSVKLTSGHLKCRLKMDKLTLKGTT 300
sw.Bali.93_LC461961       201 FYVMTVGSKSFLVHREWFHDLALPWTSSSNTAWRNRELLMEFEEAHATKQSVVALGSQEGALHQALAGAIVVEYSSSVKLTSGHLKCRLKMDKLTLKGTT 300
JKT6468_AY184212          201 FYVMTVGSKSFLVHREWFHDLALPWTSSSNTAWRNRELLMEFEEAHATKQSVVALGSQERALHQALAGAIVVEYSSSVKLTSGHLKCRLKMDKLALKGTT 300

19CxBa-83-Cv_LC579814     301 YGMCTEKFSFAKNPADTGHGTVVIELQYSGSDGPCKIPIVSVASLNDMTPVGRLVTVNPFVATSSSNSKVLVEMEPPFGDSYIVVGRGDKQINHHWHKPG 400
sw-22-00722-11.Qld_ON624132 301 YGMCTEKFSFAKNPADTGHGTVVIELQYSGSDGPCKIPIVSVASLNDMTPVGRLVTVNPFVATSSSNSKVLVEMEPPFGDSYIVVGRGDKQINHHWHKPG 400
Hu_Tiwi 2021_OM867669     301 YGMCTEKFSFAKNPADTGHGTVVIELQYSGSDGPCKIPIVSVASLNDMTPVGRLVTVNPFVATSSSNSKVLVEMEPPFGDSYIVVGRGDKQINHHWHKPG 400
Bali2019_MT253731         301 YGMCTEKFSFAKNPADTGHGTVVIELQYSGSDGPCKIPIVSVASLNDMTPVGRLVTVNPFVATSSSNSKVLVEMEPPFGDSYIVVGRGDKQINHHWHKPG 400
sw.Bali.93_LC461961       301 YGMCTEKFSFAKNPADTGHGTVVIELQYSGSDGPCKIPIVSVASLNDMTPVGRLVTVNPFVATSSSNSKVLVEMEPPFGDSYIVVGRGDKQINHHWHKPG 400
JKT6468_AY184212          301 YGMCTEKFSFAKNPADTGHGTVVIELLYSGSDGPCKIPIVSVASLNDMTPVGRLVTVNPFVATSSSNSQVLVEMEPPFGDSYIVVGRGEKQINHHWHKPG 400

19CxBa-83-Cv_LC579814     401 STLGKAFSTTLKGAQRLAALGDTAWDFGSIGGVFNSIGKAVHQVFGGAFRTLFGGMSWITQGLMGALLLWMGVNARDRSIAMAFLVTGGTLLFLATNVHA 500
sw-22-00722-11.Qld_ON624132 401 STLGKAFSTTLKGAQRLAALGDTAWDFGSIGGVFNSIGKAVHQVFGGAFRTLFGGMSWITQGLMGALLLWMGINARDRSIAVAFLVTGGTLLFLATNVHA 500
Hu_Tiwi 2021_OM867669     401 STLGKAFSTTLKGAQRLAALGDTAWDFGSIGGVFNSIGKAVHQVFGGAFRTLFGGMSWITQGLMGALLLWMGINARDRSIAVAFLVTGGTLLFLATNVHA 500
Bali2019_MT253731         401 STLGKAFSTTLKGAQRLAALGDTAWDFGSIGGVFNSIGKAVHQVFGGAFRTLFGGMSWITQGLMGALLLWMGVNARDRSIAMAFLVTGGTLLFLATNVHA 500
sw.Bali.93_LC461961       401 STLGKAFSTTLKGAQRLAALGDTAWDFGSIGGVFNSIGKAVHQVFGGAFRTLFGGMSWITQGLMGALLLWMGVNARDRSIAMAFLVTGGTLLFLATNVHA 500
JKT6468_AY184212          401 STLGKAFSTTLKGAQRLAALGDTAWDFGSIGGVFNSIGKAVHQVFGGAFRTLFGGMSWITQGLMGVLLLWMGINARDRSIAMAFLVTGGTLLFLATNVHA 500
```

Figure S3. Comparison of the complete amino acid sequence of E protein (500 residues) in GIV JEV strains.

Figure S4

```
I_Mie41          1 FNCLGMGNRDFIEGASGATWVDLVLEGDSCLTIMANDKPTLDVRMINIEASQLAEVRSYCYHASVTDISTVARCPTTGEAHNEKRADSSYVCKQGFTDRG 100
III_Beijing-1    1 FNCLGMGNRDFIEGASGATWVDLVLEGDSCLTIMANDKPTLDVRMINIEASQLAEVRSYCYHASVTDISTVARCPTTGEAHNEKRADSSYVCKQGFTDRG 100
IV_19CxBa-83     1 FNCLGMGNRDFIEGVSGATWVDLVLEGDSCLTIMAHDRPTLDVRMVNIEASQLAEVRSYCYHASVTDISTVARCPTTGEAHNDKRADSNYVCKQGFTDRG 100
V_Muar           1 FNCLGMGNRDFIEGVSGATWVDLVLEGDSCLTIMANDKPTLDVRMINIEATQLAEVRTYCYHATVADISTVARCPTTGEAHNTRRADSSYVCKQGYTDRG 100

I_Mie41        101 WGNGCGLFGKGSIDTCAKFSCTSKAIGRMIQPENIKYEVGIFVHGTTTSENHGNYSAQVGASQAAKFTVTPNAPSITLKLGDYGEVTLDCEPRSGLNTEA 200
III_Beijing-1  101 WGNGCGLFGKGSIDTCAKFSCTSKAIGRTIQSENIKYEVGIFVHGTTTSENHGNYSAQVGASQAAKFTVTPNAPSITLKLGDYGEVTLDCEPRSGLNTEA 200
IV_19CxBa-83   101 WGNGCGLFGKGSIDTCAKFSCTSKATGKTIQPENIKYEVGIFVHGTTTSENHGNYTAQIGASQAAKFTITPNAPSITLKLGDYGEVTLDCEPRSGLNTEA 200
V_Muar         101 WGNGCGLFGKGSIDTCAKFVCSHKAIGKIIQPENIKYEVGVFVHGTTTAENHGNYSAQIGASQAAKFTITPNAPSITLKLGDYGEVTMDCEPRSGFNTEA 200

I_Mie41        201 FYVMTVGSKSFLVHREWFHDLSLPWTSPSSTAWRNRELLMEFEEAHATKQSVVALGSQEGGLHQALAGAIVVEYSSSVKLTSGHLKCRLKMDKLALKGTT 300
III_Beijing-1  201 FYVMTVGSKSFLVHREWFHDLALPWTPPSSTAWRNRELLMEFEEAHATKQSVVALGSQEGGLHQALAGAIVVEYSSSVKLTSGHLKCRLKMDKLALKGTT 300
IV_19CxBa-83   201 FYVMTVGSKSFLVHREWFHDLALPWTSASNTAWRNRELLMEFEEAHATKQSVVALGSQEGALHQALAGAIVVEYSSSVKLTSGHLKCRLKMDKLTLKGTT 300
V_Muar         201 FYVLTVGTKSFLVHREWFNDLALPWLSPSSTNWRNREILLEFEEAHATKQSVVALGSQEGALHQALAGAIVVEYSSSVKLTSGHLKCRLKMDKLALKGTT 300

I_Mie41        301 YGMCTEKFSFAKNPADTGHGTVVIELTYSGSDGPCKIPIVSVASLNDMTPVGRLVTVNPFVATSSSNSKVLVEMEPPFGDSYIVVGRGDKQINHHWHKAG 400
III_Beijing-1  301 YGMCTEKFSFAKNPADTGHGTVVIELSYSGSDGPCKIPIVSVASLNDMTPVGRLVTVNPFVATSSANSKVLVEMEPPFGDSYIVVGRGDKQINHHWYKAG 400
IV_19CxBa-83   301 YGMCTEKFSFAKNPADTGHGTVVIELQYSGSDGPCKIPIVSVASLNDMTPVGRLVTVNPFVATSSSNSKVLVEMEPPFGDSYIVVGRGDKQINHHWHKPG 400
V_Muar         301 YGMCTEKFSFSKNPADTGHGTVVIELQYTGTDGPCKIPISSVASLNDLTPVGRLVTVNPFVATSTANSKVLVELEPPFGDSFIVVGRGDKQINHHWHKAG 400

I_Mie41        401 STLGKAFSTTLKGAQRLAALGDTAWDFGSIGGVFNSIGKAVHQVFGGAFRTLFGGMSWITQGLMGALLLWMGVNARDRSIALAFLATGGVLVFLATNVHA 500
III_Beijing-1  401 STLGKAFSTTLKGAQRLAALGDTAWDFGSIGGVFNSIGKAVHQVFGGAFRTLFGGMSWITQGLMGALLLWMGINARDRSIALAFLATGGVLVFLATNVHA 500
IV_19CxBa-83   401 STLGKAFSTTLKGAQRLAALGDTAWDFGSIGGVFNSIGKAVHQVFGGAFRTLFGGMSWITQGLMGALLLWMGVNARDRSIAMAFLVTGGTLLFLATNVHA 500
V_Muar         401 SSLGKAFTTTLKGAQRLAALGDTAWDFGSIGGVFNSIGKAVHQVFGGAFRTLFGGMSWITQGLMGALLLWMGINARDRSIALAFLATGGVLLFLATNVHA 500
```

Strain vs. strain	AA identity
Mie/41/2002 vs. 19CxBa-83-Cv	95.4%
Mie/41/2002 vs. Muar	91.6%
19CxBa-83-Cv vs. Muar	90.6%

Strain	AA difference (vs. GIII_Beijing-1)
Mie/41/2002	8/500 (1.6%)
19CxBa-83-Cv	26/500 (5.2%)
Muar	42/500 (8.4%)

Figure S4. Comparison of the complete amino acid sequence of E protein (500 residues) of Mie/41/2002 (GI), Beijing-1 (GIII, accession no. L48961), 19CxBa-83-Cv (GIV), and Muar (GV) strains of JEV.
